# Crystal structure of bis­[2-(1*H*-benzimidazol-2-yl-κ*N* ^3^)aniline-κ*N*]bis­(nitrato-κ*O*)cadmium(II)

**DOI:** 10.1107/S2056989019012416

**Published:** 2019-09-12

**Authors:** Yongtae Kim, Sung Kwon Kang

**Affiliations:** a Department of Chemistry, Chungnam National University, Daejeon 305-764, Republic of Korea

**Keywords:** crystal structure, Cd^II^*N*-heterocyclic complex, benzimidazole, N—H⋯O hydrogen bonds

## Abstract

In the title compound, [Cd(NO_3_)_2_(C_13_H_11_N_3_)_2_], the Cd^II^ atom lies on a twofold rotation axis and is coordinated by four N atoms and two O atoms, provided by two bidentate 2-(1*H*-benzimidazol-2-yl)aniline ligands, and two nitrato O atoms, forming a distorted octa­hedral geometry [range of bond angles around the Cd atom = 73.82 (2)–106.95 (8)°]. In the ligand, the dihedral angle between the aniline ring and the benzimidazole ring system is 30.43 (7)°. The discrete complex mol­ecule is stabilized by an intra­molecular N—H⋯O hydrogen bond. In the crystal, inter­molecular N—H⋯O hydrogen bonds link the mol­ecules, forming a three-dimensional network.

## Abstract 2

The synthesis and crystal structure of the title 2-(1*H*-benzimidazol-2-yl)aniline Cd^II^ complex is reported in which the Cd^II^ atom lies on a twofold rotation axis and is coordinated by four N atoms, provided by two bidentate 2-(1*H*-benzimidazol-2-yl)aniline ligands, and two nitrato O atoms, forming a distorted octa­hedral geometry.

## Chemical context   

Azole and benzazole derivatives are well-known nitro­gen-containing heterocyclic compounds, and are of great inter­est because of their broad spectrum of biological activity (Esparza-Ruiz *et al.*, 2011 ▸; Hock *et al.*, 2013 ▸). Imidazole is an aza­pyrrole in which the nitro­gen atoms are separated by one carbon atom. Benzimidazole, a fused heterocycle with benzene and imidazole, is associated with a wide array of pharmacological activities (Akhtar *et al.*, 2017 ▸), and benzimidazole derivatives exhibit a wide range of various biological activities. These include bactericidal (Carcanague *et al.*, 2002 ▸) and fungicidal (Lezcano *et al.*, 2002 ▸; Aghatabay *et al.*, 2007 ▸) properties. Their metal complexes have been shown to display anti­tumor activity and are important biological mol­ecules (Sánchez-Guadarrama *et al.*, 2009 ▸; Ramla *et al.*, 2007 ▸; Wang *et al.*, 2007 ▸). Recently, we reported on the synthesis and structural features of Zn (Kim & Kang, 2015*a* ▸) and Ag (Kim & Kang, 2015*b* ▸) complexes with benzimidazole derivatives. In this work, we have synthesized the title compound and characterized it by single crystal X-ray crystallography.
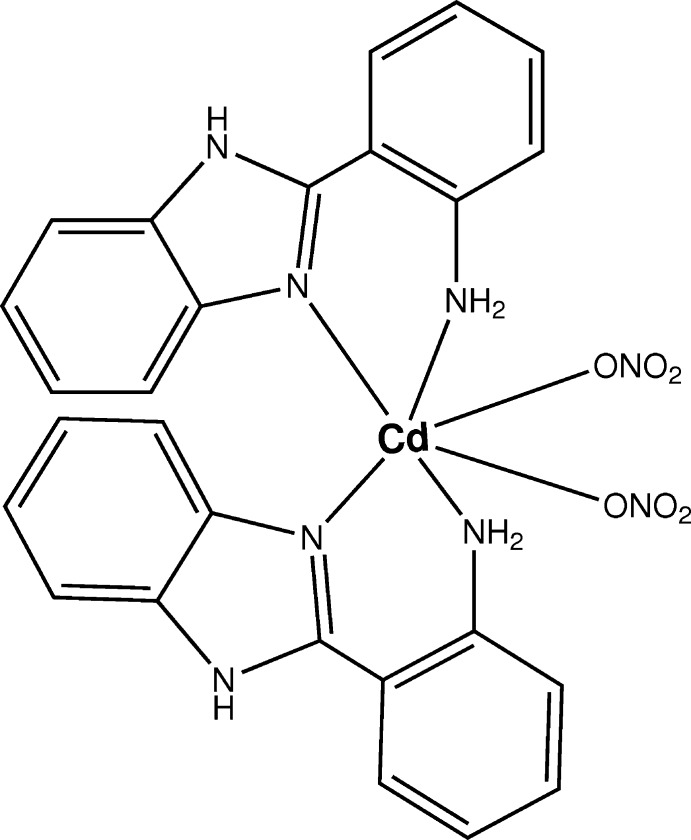


## Structural commentary   

**Figure 1:**
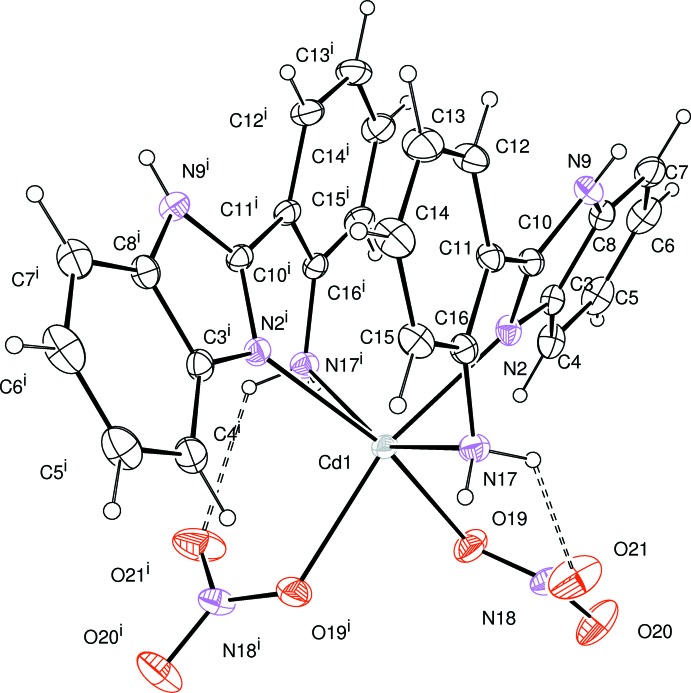
Mol­ecular structure of the title compound, showing the atom-numbering scheme and displacement ellipsoids drawn at the 30% probability level. The intramolecular N—H⋯O hydrogen bonds are indicated by dashed lines. [Symmetry code: (i) −*x* + 1, *y*, −*z* + 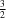.]

The mol­ecular structure of the title complex is shown in Fig. 1 ▸. The complex lies about a twofold rotation axis which passes through the Cd^II^ atom, the coordination geometry around which is distorted octa­hedral with two O atoms of two nitrato ligands and four N atoms of two bidentate 2-(1*H*-benzimidazol-2-yl)aniline ligands. The Cd—N and Cd—O bond lengths [Cd1—N2 = 2.317 (2), Cd1—N17 = 2.437 (2) and Cd1—O19 = 2.3175 (19) Å] are comparable with those of other Cd complexes (Barszcz *et al.*, 2013 ▸; Jalilehvand *et al.*, 2009 ▸). The bond angles around the Cd1 atom are in the range of 73.82 (8)–106.95 (8)°. The dihedral angle between the benzimidazole (N2/C3–C8/N9/C10) ring system and the aniline (C11–C16/N17) plane in the bidentate ligand is 30.43 (7)°. This twisting is a driving force in the formation of weak Cd1—N17 bonding, this bond being [2.437 (2) Å] a little longer than Cd1—N2 [2.317 (2) Å]. This elongation was also observed in our previous studies of imidazole­aniline–metal complexes (Zn: Kim & Kang, 2015*a* ▸; Ag: Kim & Kang, 2015*b* ▸). The N2—C10 bond length of 1.327 (3) Å in the imidazole ring shows double-bond character compared to the other N—C bond lengths [N2—C3 = 1.397 (3), C8—N9 = 1.384 (3) and N9—C10 = 1.355 (3) Å]. The discrete mol­ecule is stabilized by an intra­molecular N—H⋯O hydrogen bond (Table 1 ▸).

## Supra­molecular features   

**Figure 2:**
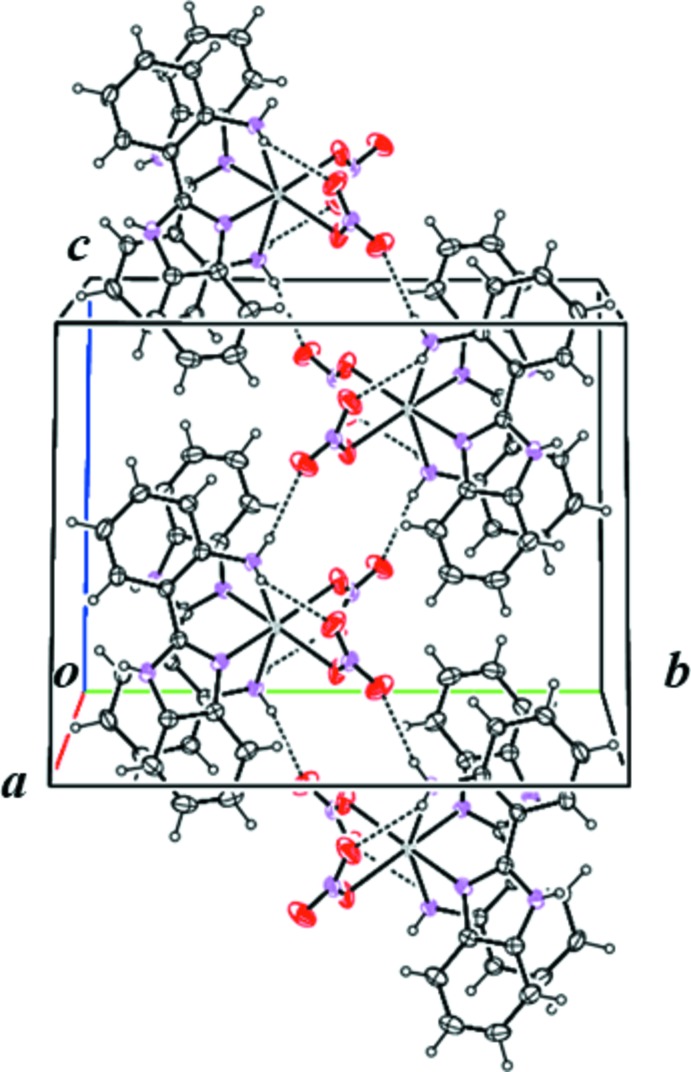
Partial packing diagram of the title compound, showing mol­ecules linked by inter­molecular N—H⋯O hydrogen bonds (dashed lines), viewed along the *a*-axis direction.

**Figure 3:**
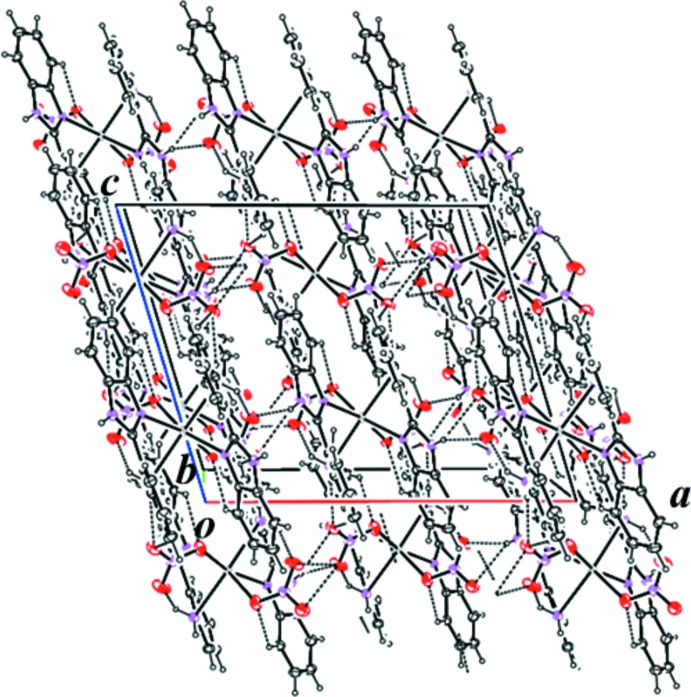
A view along the *b* axis of the crystal packing of the title compound, showing the three-dimensional network linked of molecules linked by N—H⋯O hydrogen bonds (dashed lines, Table 1 ▸).

In the crystal, mol­ecules are linked by a series of N—H⋯O inter­actions. The nitrate group containing oxygen atom O21 forms both intra- and inter­molecular hydrogen bonds. Mol­ecules are arranged into a zigzag chain along the *c*-axis direction *via* an N—H⋯O hydrogen bond (N17—H17*B*⋯O20^ii^; symmetry code as in Table 1 ▸; Fig. 2 ▸). The other N—H⋯O hydrogen bonds (N9—H9⋯O20^i^ and N9—H9⋯O21^i^; Table 1 ▸) link the mol­ecules into a three-dimensional network (Fig. 3 ▸).

## Database survey   

A search of the Cambridge Structural Database (CSD, Version 5.40, Feb. 2019; Groom *et al.*, 2016 ▸) gave 4678 entries for crystal structures related to benzimidazoles. However, there are only 14 entries involving the ligands 2-(1*H*-benzimidazol-2-yl)aniline or 2-(2-amino­phen­yl)-1*H*-benzimidazole with a transition metal. These include Ni (refcode EWUZOM; Esparza-Ruiz *et al.*, 2011 ▸), Zn [AWOLEE (Eltayeb *et al.*, 2011 ▸) and JUFCOE (Kim & Kang, 2015*a* ▸)], Ru (NUNLID; Małecki, 2012 ▸) and Re (UYELEQ; Machura *et al.*, 2011 ▸).

## Synthesis and crystallization   

Chemicals were obtained commercially in reagent grade and used as received. Solvents were dried using standard procedures described in the literature. To a stirred solution of Cd(NO_3_)·4H_2_O (0.154 g, 0.5 mmol) in ethanol (20 ml) was added a solution of 2-(1*H*-benzimidazol-2-yl)aniline (0.209 g, 1.0 mmol) in ethanol (10 ml) at 333 K. After 24 h of stirring, the title complex was obtained as a white powder. The powder was filtered off and washed with ethanol. Colourless crystals of the title complex were obtained by slow evaporation of the methanol solvent at room temperature within two weeks.

## Refinement   

Crystal data, data collection and structure refinement details are summarized in Table 2 ▸. H atoms of the NH and NH_2_ groups were located in a difference-Fourier map and refined freely [refined distances: N—H = 0.75 (3)–0.86 (3) Å]. Other H atoms were positioned geometrically and refined using a riding model, with C—H = 0.93 Å, and with *U*_iso_(H) = 1.2*U*_eq_(C).

## Supplementary Material

Crystal structure: contains datablock(s) I. DOI: 10.1107/S2056989019012416/is5523sup1.cif

Structure factors: contains datablock(s) I. DOI: 10.1107/S2056989019012416/is5523Isup2.hkl

CCDC reference: 1951821

Additional supporting information:  crystallographic information; 3D view; checkCIF report

## Figures and Tables

**Table 1:** Hydrogen-bond geometry (Å, °)

*D*—H⋯*A*	*D*—H	H⋯*A*	*D*⋯*A*	*D*—H⋯*A*
N9—H9⋯O20^i^	0.75 (3)	2.39 (3)	3.012 (3)	140 (3)
N9—H9⋯O21^i^	0.75 (3)	2.51 (3)	3.238 (3)	163 (3)
N17—H17*A*⋯O21	0.86 (3)	2.34 (3)	2.973 (3)	131 (2)
N17—H17*B*⋯O20^ii^	0.79 (3)	2.24 (3)	3.024 (3)	170 (3)

Symmetry codes: (i) 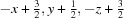; (ii) 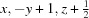.

**Table 2:** Experimental details

Crystal data	
Chemical formula	[Cd(NO_3_)_2_(C_13_H_11_N_3_)_2_]
*M*_r_	654.91
Crystal system, space group	Monoclinic, *C*2/*c*
Temperature (K)	296
*a*, *b*, *c* (Å)	14.6899 (4), 15.0250 (3), 12.2269 (3)
β (°)	106.8431 (15)
*V* (Å^3^)	2582.90 (11)
*Z*	4
Radiation type	Mo *K*α
μ (mm^−1^)	0.91
Crystal size (mm)	0.15 × 0.13 × 0.12

Data collection	
Diffractometer	Bruker *SMART* CCD area-detector
Absorption correction	Multi-scan (*SADABS*; Bruker, 2012 ▸)
*T*_min_, *T*_max_	0.546, 0.726
No. of measured, independent and observed [*I* > 2σ(*I*)] reflections	11727, 3087, 2729
*R*_int_	0.031
(sin θ/λ)_max_ (Å^−1^)	0.667

Refinement	
*R*[*F* ^2^ > 2σ(*F* ^2^)], *wR*(*F* ^2^), *S*	0.035, 0.078, 1.06
No. of reflections	3087
No. of parameters	198
H-atom treatment	H atoms treated by a mixture of independent and constrained refinement
Δρ_max_, Δρ_min_ (e Å^−3^)	0.82, −0.35

Computer programs: *SMART* and *SAINT* (Bruker, 2012 ▸), *SHELXS2013* (Sheldrick, 2008 ▸), *SHELXL2013* (Sheldrick, 2015 ▸) and *ORTEP-3 for Windows* and *WinGX* (Farrugia, 2012 ▸).

## supplementary crystallographic information

### Crystal data

**Table:**

[Cd(NO_3_)_2_(C_13_H_11_N_3_)_2_]	*F*(000) = 1320
*M**_r_* = 654.91	*D*_x_ = 1.684 Mg m^−^^3^
Monoclinic, *C*2/*c*	Mo *K*α radiation, λ = 0.71073 Å
*a* = 14.6899 (4) Å	Cell parameters from 5554 reflections
*b* = 15.0250 (3) Å	θ = 2.4–28.0°
*c* = 12.2269 (3) Å	µ = 0.91 mm^−^^1^
β = 106.8431 (15)°	*T* = 296 K
*V* = 2582.90 (11) Å^3^	Block, colourless
*Z* = 4	0.15 × 0.13 × 0.12 mm

### Data collection

**Table:**

Bruker SMART CCD area-detector diffractometer	2729 reflections with *I* > 2σ(*I*)
Radiation source: fine-focus sealed tube	*R*_int_ = 0.031
φ and ω scans	θ_max_ = 28.3°, θ_min_ = 2.0°
Absorption correction: multi-scan (SADABS; Bruker, 2012)	*h* = −19→17
*T*_min_ = 0.546, *T*_max_ = 0.726	*k* = −19→20
11727 measured reflections	*l* = −16→16
3087 independent reflections	

### Refinement

**Table:**

Refinement on *F*^2^	0 restraints
Least-squares matrix: full	Hydrogen site location: mixed
*R*[*F*^2^ > 2σ(*F*^2^)] = 0.035	H atoms treated by a mixture of independent and constrained refinement
*wR*(*F*^2^) = 0.078	*w* = 1/[σ^2^(*F*_o_^2^) + (0.0422*P*)^2^ + 0.8298*P*] where *P* = (*F*_o_^2^ + 2*F*_c_^2^)/3
*S* = 1.06	(Δ/σ)_max_ = 0.001
3087 reflections	Δρ_max_ = 0.82 e Å^−^^3^
198 parameters	Δρ_min_ = −0.35 e Å^−^^3^

### Special details

**Table:**

Geometry. All esds (except the esd in the dihedral angle between two l.s. planes) are estimated using the full covariance matrix. The cell esds are taken into account individually in the estimation of esds in distances, angles and torsion angles; correlations between esds in cell parameters are only used when they are defined by crystal symmetry. An approximate (isotropic) treatment of cell esds is used for estimating esds involving l.s. planes.

### Fractional atomic coordinates and isotropic or equivalent isotropic displacement parameters (Å^2^)

**Table:**

	*x*	*y*	*z*	*U*_iso_*/*U*_eq_	
Cd1	0.5000	0.62214 (2)	0.7500	0.03198 (10)	
N2	0.59443 (14)	0.72062 (13)	0.68561 (17)	0.0345 (4)	
C3	0.60535 (16)	0.72958 (16)	0.5763 (2)	0.0357 (5)	
C4	0.57954 (19)	0.6718 (2)	0.4835 (2)	0.0470 (7)	
H4	0.5507	0.6173	0.4877	0.056*	
C5	0.5990 (2)	0.6994 (2)	0.3847 (3)	0.0562 (8)	
H5	0.5826	0.6628	0.3206	0.067*	
C6	0.6425 (2)	0.7807 (2)	0.3793 (3)	0.0594 (8)	
H6	0.6538	0.7970	0.3110	0.071*	
C7	0.6695 (2)	0.8379 (2)	0.4705 (3)	0.0495 (7)	
H7	0.6997	0.8917	0.4663	0.059*	
C8	0.64909 (17)	0.81075 (17)	0.5695 (2)	0.0373 (5)	
N9	0.66350 (16)	0.85021 (15)	0.67542 (19)	0.0380 (5)	
H9	0.696 (2)	0.889 (2)	0.697 (3)	0.054 (10)*	
C10	0.62989 (16)	0.79434 (15)	0.7417 (2)	0.0323 (5)	
C11	0.62624 (16)	0.81778 (15)	0.8566 (2)	0.0334 (5)	
C12	0.6159 (2)	0.90724 (18)	0.8829 (2)	0.0443 (6)	
H12	0.6155	0.9506	0.8285	0.053*	
C13	0.6065 (2)	0.93253 (19)	0.9865 (3)	0.0540 (7)	
H13	0.5984	0.9922	1.0017	0.065*	
C14	0.6090 (2)	0.86845 (19)	1.0683 (3)	0.0520 (7)	
H14	0.6028	0.8852	1.1390	0.062*	
C15	0.62043 (18)	0.78054 (18)	1.0463 (2)	0.0428 (6)	
H15	0.6230	0.7383	1.1027	0.051*	
C16	0.62832 (16)	0.75354 (16)	0.9400 (2)	0.0331 (5)	
N17	0.63122 (16)	0.66172 (14)	0.9164 (2)	0.0367 (5)	
H17A	0.674 (2)	0.6453 (18)	0.885 (3)	0.040 (8)*	
H17B	0.640 (2)	0.6314 (18)	0.972 (3)	0.037 (8)*	
N18	0.63805 (17)	0.48486 (14)	0.6968 (2)	0.0457 (5)	
O19	0.55302 (15)	0.50697 (13)	0.6581 (2)	0.0584 (6)	
O20	0.67211 (17)	0.43131 (17)	0.6449 (2)	0.0793 (8)	
O21	0.6875 (2)	0.51407 (17)	0.7877 (2)	0.0861 (8)	

### Atomic displacement parameters (Å^2^)

**Table:**

	*U*^11^	*U*^22^	*U*^33^	*U*^12^	*U*^13^	*U*^23^
Cd1	0.02785 (14)	0.02431 (13)	0.04112 (16)	0.000	0.00582 (10)	0.000
N2	0.0292 (11)	0.0388 (11)	0.0348 (11)	−0.0043 (8)	0.0083 (8)	−0.0076 (9)
C3	0.0269 (12)	0.0433 (14)	0.0362 (14)	0.0012 (9)	0.0078 (10)	−0.0038 (11)
C4	0.0370 (15)	0.0591 (18)	0.0432 (16)	−0.0004 (12)	0.0090 (11)	−0.0138 (13)
C5	0.0521 (18)	0.076 (2)	0.0366 (16)	0.0124 (15)	0.0060 (13)	−0.0119 (15)
C6	0.066 (2)	0.079 (2)	0.0350 (16)	0.0263 (17)	0.0172 (14)	0.0110 (16)
C7	0.0522 (18)	0.0522 (17)	0.0479 (17)	0.0117 (13)	0.0205 (13)	0.0133 (14)
C8	0.0321 (13)	0.0434 (14)	0.0361 (14)	0.0049 (10)	0.0092 (10)	0.0016 (11)
N9	0.0379 (12)	0.0354 (11)	0.0402 (12)	−0.0066 (9)	0.0107 (9)	−0.0014 (10)
C10	0.0267 (12)	0.0323 (12)	0.0351 (13)	−0.0033 (9)	0.0046 (9)	−0.0005 (10)
C11	0.0320 (13)	0.0318 (12)	0.0355 (13)	−0.0066 (9)	0.0084 (10)	−0.0045 (10)
C12	0.0547 (17)	0.0322 (12)	0.0476 (16)	−0.0077 (11)	0.0175 (13)	−0.0027 (12)
C13	0.070 (2)	0.0368 (15)	0.0595 (19)	−0.0068 (13)	0.0257 (16)	−0.0164 (14)
C14	0.066 (2)	0.0513 (17)	0.0434 (16)	−0.0139 (14)	0.0238 (14)	−0.0129 (13)
C15	0.0465 (16)	0.0441 (15)	0.0365 (15)	−0.0082 (11)	0.0097 (11)	−0.0019 (11)
C16	0.0265 (12)	0.0341 (12)	0.0357 (13)	−0.0047 (9)	0.0042 (9)	−0.0031 (10)
N17	0.0372 (13)	0.0329 (11)	0.0374 (13)	0.0007 (9)	0.0068 (10)	0.0019 (10)
N18	0.0556 (15)	0.0329 (11)	0.0444 (14)	0.0093 (10)	0.0079 (11)	−0.0038 (10)
O19	0.0457 (12)	0.0411 (11)	0.0832 (16)	0.0077 (8)	0.0105 (11)	−0.0139 (10)
O20	0.0740 (16)	0.0897 (19)	0.0650 (15)	0.0407 (14)	0.0059 (12)	−0.0261 (14)
O21	0.099 (2)	0.0681 (16)	0.0665 (17)	0.0171 (14)	−0.0153 (14)	−0.0294 (13)

### Geometric parameters (Å, º)

**Table:**

Cd1—N2	2.317 (2)	N9—C10	1.355 (3)
Cd1—N2^i^	2.317 (2)	N9—H9	0.75 (3)
Cd1—O19	2.3175 (19)	C10—C11	1.464 (3)
Cd1—O19^i^	2.3175 (19)	C11—C16	1.398 (3)
Cd1—N17	2.437 (2)	C11—C12	1.401 (3)
Cd1—N17^i^	2.437 (2)	C12—C13	1.368 (4)
N2—C10	1.327 (3)	C12—H12	0.9300
N2—C3	1.397 (3)	C13—C14	1.381 (4)
C3—C4	1.392 (4)	C13—H13	0.9300
C3—C8	1.392 (3)	C14—C15	1.368 (4)
C4—C5	1.384 (4)	C14—H14	0.9300
C4—H4	0.9300	C15—C16	1.398 (4)
C5—C6	1.389 (5)	C15—H15	0.9300
C5—H5	0.9300	C16—N17	1.413 (3)
C6—C7	1.373 (4)	N17—H17A	0.86 (3)
C6—H6	0.9300	N17—H17B	0.79 (3)
C7—C8	1.390 (4)	N18—O20	1.218 (3)
C7—H7	0.9300	N18—O21	1.219 (3)
C8—N9	1.384 (3)	N18—O19	1.246 (3)
			
N2—Cd1—N2^i^	100.60 (10)	C7—C8—C3	122.0 (3)
N2—Cd1—O19	89.64 (8)	C10—N9—C8	108.1 (2)
N2^i^—Cd1—O19	163.78 (7)	C10—N9—H9	125 (3)
N2—Cd1—O19^i^	163.78 (7)	C8—N9—H9	125 (3)
N2^i^—Cd1—O19^i^	89.64 (8)	N2—C10—N9	111.4 (2)
O19—Cd1—O19^i^	83.39 (11)	N2—C10—C11	125.3 (2)
N2—Cd1—N17	73.82 (8)	N9—C10—C11	123.1 (2)
N2^i^—Cd1—N17	88.10 (7)	C16—C11—C12	118.4 (2)
O19—Cd1—N17	106.95 (8)	C16—C11—C10	122.3 (2)
O19^i^—Cd1—N17	94.19 (8)	C12—C11—C10	119.2 (2)
N2—Cd1—N17^i^	88.10 (7)	C13—C12—C11	121.8 (3)
N2^i^—Cd1—N17^i^	73.82 (8)	C13—C12—H12	119.1
O19—Cd1—N17^i^	94.18 (8)	C11—C12—H12	119.1
O19^i^—Cd1—N17^i^	106.95 (8)	C12—C13—C14	119.2 (3)
N17—Cd1—N17^i^	151.75 (10)	C12—C13—H13	120.4
C10—N2—C3	106.1 (2)	C14—C13—H13	120.4
C10—N2—Cd1	122.79 (16)	C15—C14—C13	120.6 (3)
C3—N2—Cd1	129.23 (15)	C15—C14—H14	119.7
C4—C3—C8	121.3 (2)	C13—C14—H14	119.7
C4—C3—N2	129.8 (2)	C14—C15—C16	120.7 (3)
C8—C3—N2	109.0 (2)	C14—C15—H15	119.6
C5—C4—C3	116.7 (3)	C16—C15—H15	119.6
C5—C4—H4	121.7	C15—C16—C11	119.2 (2)
C3—C4—H4	121.7	C15—C16—N17	119.2 (2)
C4—C5—C6	121.3 (3)	C11—C16—N17	121.4 (2)
C4—C5—H5	119.3	C16—N17—Cd1	110.21 (15)
C6—C5—H5	119.3	C16—N17—H17A	116.2 (19)
C7—C6—C5	122.7 (3)	Cd1—N17—H17A	93 (2)
C7—C6—H6	118.6	C16—N17—H17B	113 (2)
C5—C6—H6	118.6	Cd1—N17—H17B	118 (2)
C6—C7—C8	116.1 (3)	H17A—N17—H17B	105 (3)
C6—C7—H7	122.0	O20—N18—O21	119.1 (3)
C8—C7—H7	122.0	O20—N18—O19	119.7 (2)
N9—C8—C7	132.5 (3)	O21—N18—O19	121.1 (2)
N9—C8—C3	105.5 (2)	N18—O19—Cd1	117.12 (17)
			
C10—N2—C3—C4	−179.5 (3)	C8—N9—C10—N2	0.1 (3)
Cd1—N2—C3—C4	−15.2 (4)	C8—N9—C10—C11	−174.8 (2)
C10—N2—C3—C8	0.4 (3)	N2—C10—C11—C16	31.7 (4)
Cd1—N2—C3—C8	164.74 (16)	N9—C10—C11—C16	−154.1 (2)
C8—C3—C4—C5	−0.4 (4)	N2—C10—C11—C12	−145.3 (2)
N2—C3—C4—C5	179.5 (3)	N9—C10—C11—C12	28.9 (4)
C3—C4—C5—C6	0.3 (4)	C16—C11—C12—C13	−1.0 (4)
C4—C5—C6—C7	0.6 (5)	C10—C11—C12—C13	176.1 (3)
C5—C6—C7—C8	−1.2 (4)	C11—C12—C13—C14	1.3 (5)
C6—C7—C8—N9	−178.7 (3)	C12—C13—C14—C15	−0.2 (5)
C6—C7—C8—C3	1.1 (4)	C13—C14—C15—C16	−1.0 (5)
C4—C3—C8—N9	179.5 (2)	C14—C15—C16—C11	1.3 (4)
N2—C3—C8—N9	−0.4 (3)	C14—C15—C16—N17	−174.2 (3)
C4—C3—C8—C7	−0.3 (4)	C12—C11—C16—C15	−0.2 (3)
N2—C3—C8—C7	179.8 (2)	C10—C11—C16—C15	−177.2 (2)
C7—C8—N9—C10	180.0 (3)	C12—C11—C16—N17	175.1 (2)
C3—C8—N9—C10	0.2 (3)	C10—C11—C16—N17	−1.9 (3)
C3—N2—C10—N9	−0.3 (3)	C15—C16—N17—Cd1	120.8 (2)
Cd1—N2—C10—N9	−165.88 (16)	C11—C16—N17—Cd1	−54.5 (3)
C3—N2—C10—C11	174.4 (2)	O20—N18—O19—Cd1	−172.3 (2)
Cd1—N2—C10—C11	8.8 (3)	O21—N18—O19—Cd1	9.7 (3)

Symmetry code: (i) −*x*+1, *y*, −*z*+3/2.

### Hydrogen-bond geometry (Å, º)

**Table:**

*D*—H···*A*	*D*—H	H···*A*	*D*···*A*	*D*—H···*A*
N9—H9···O20^ii^	0.75 (3)	2.39 (3)	3.012 (3)	140 (3)
N9—H9···O21^ii^	0.75 (3)	2.51 (3)	3.238 (3)	163 (3)
N17—H17*A*···O21	0.86 (3)	2.34 (3)	2.973 (3)	131 (2)
N17—H17*B*···O20^iii^	0.79 (3)	2.24 (3)	3.024 (3)	170 (3)

Symmetry codes: (ii) −*x*+3/2, *y*+1/2, −*z*+3/2; (iii) *x*, −*y*+1, *z*+1/2.
